# Genomic Characterization of Multidrug-Resistant *Escherichia coli* BH100 Sub-strains

**DOI:** 10.3389/fmicb.2020.549254

**Published:** 2021-01-08

**Authors:** Rodrigo Carvalho, Flavia Aburjaile, Marcus Canario, Andréa M. A. Nascimento, Edmar Chartone-Souza, Luis de Jesus, Andrey A. Zamyatnin, Bertram Brenig, Debmalya Barh, Preetam Ghosh, Aristoteles Goes-Neto, Henrique C. P. Figueiredo, Siomar Soares, Rommel Ramos, Anne Pinto, Vasco Azevedo

**Affiliations:** ^1^Institute of Molecular Medicine, Sechenov First Moscow State Medical University, Moscow, Russia; ^2^Departamento de Genética, Ecologia e Evolução, Universidade Federal de Minas Gerais, Belo Horizonte, Brazil; ^3^Departamento de Genética, Universidade Federal de Pernambuco, Recife, Brazil; ^4^Belozersky Institute of Physico-Chemical Biology, Lomonosov Moscow State University, Moscow, Russia; ^5^Institute of Veterinary Medicine, University of Göttingen, Göttingen, Germany; ^6^Institute of Integrative Omics and Applied Biotechnology, Purba Medinipur, India; ^7^Department of Computer Science, Virginia Commonwealth University, Richmond, VA, United States; ^8^Departmento de Microbiologia, Imunologia e Parasitologia, Universidade Federal do Triangulo Mineiro, Uberaba, Brazil; ^9^Universidade Federal do Pará, Belém, Brazil

**Keywords:** antibiotic resistance, genomic sequencing, mobile genetic elements, urinary tract infection, UPEC

## Abstract

The rapid emergence of multidrug-resistant (MDR) bacteria is a global health problem. Mobile genetic elements like conjugative plasmids, transposons, and integrons are the major players in spreading resistance genes in uropathogenic *Escherichia coli* (UPEC) pathotype. The *E. coli* BH100 strain was isolated from the urinary tract of a Brazilian woman in 1974. This strain presents two plasmids carrying MDR cassettes, pBH100, and pAp, with conjugative and mobilization properties, respectively. However, its transposable elements have not been characterized. In this study, we attempted to unravel the factors involved in the mobilization of virulence and drug-resistance genes by assessing genomic rearrangements in four BH100 sub-strains (BH100 MG2014, BH100 MG2017, BH100L MG2017, and BH100N MG2017). Therefore, the complete genomes of the BH100 sub-strains were achieved through Next Generation Sequencing and submitted to comparative genomic analyses. Our data shows recombination events between the two plasmids in the sub-strain BH100 MG2017 and between pBH100 and the chromosome in BH100L MG2017. In both cases, *IS3* and *IS21* elements were detected upstream of *Tn21* family transposons associated with MDR genes at the recombined region. These results integrated with Genomic island analysis suggest pBH100 might be involved in the spreading of drug resistance through the formation of resistance islands. Regarding pathogenicity, our results reveal that BH100 strain is closely related to UPEC strains and contains many *IS3* and *IS21-*transposase-enriched genomic islands associated with virulence. This study concludes that those IS elements are vital for the evolution and adaptation of BH100 strain.

## Introduction

*Escherichia coli* are Gram-negative bacteria naturally found in the intestinal tract of several animal species and humans, where some strains can coexist with the host in a symbiotic relationship while others may cause disease (Moulin-Schouleur et al., 2007; Römer et al., 2012; Poolman and Wacker, 2016; Kittana et al., 2018). A subset of *E. coli* strains is capable of causing extra-intestinal disease, including urinary tract infection (UTI), in which the pathotype uropathogenic *E. coli* (UPEC) accounts for 90% of global cases (Marrs et al., 2005). In this context, multidrug-resistant (MDR) strains have been considered as a major bottleneck limiting the effective control of infections. Indeed, the abuse of antibiotics has contributed to the selection of strains with the ability to acquire drug-resistance genes via horizontal transfer mechanisms (Davies and Davies, 2010; Leungtongkam et al., 2018). Previous studies suggest that conjugative R plasmids and integrons are highly prevalent in UPEC isolates and are likely maintained in bacterial populations under the selective pressure of antimicrobials (Oliveira-Pinto et al., 2017).

In the last decade, Whole-genome sequencing (WGS) approaches have been considered as valuable tools to improve the understanding of *E. coli* populations and the relationship between strains and clinical significance (Klemm and Dougan, 2016; Nakano et al., 2019). *E. coli* BH100 was isolated in the city of Belo Horizonte in 1974 from urine samples from women with UTI (Chartone-Souza, 2017). This bacterium is a non-colicin producer, lactose fermenter (lac +) and harbors two plasmids, pBH100 and pAp, of approximately 60 and 10 MDa respectively. Previous studies have focused on the phenotype characterization of these strains based on antibiotic susceptibility and plasmid transference experiments. It has been shown that pBH100 presents conjugative properties and is responsible for the resistance to inorganic mercury, chloramphenicol, kanamycin, tetracycline, and potential resistance to streptomycin. Furthermore, the smaller plasmid, pAp, can be mobilized by pBH100 and seems to provide resistance to β-lactams (Nascimento et al., 1992; Nascimento and Chartone-Souza, 2003). These previous phenotyping and molecular approaches allowed the description of the individual and small clusters of resistance elements. However, a WGS approach of this strain is required to provide a comprehensive investigation of the diversity and evolutionary relationship of *E. coli* BH100. Interestingly, the long-term cultivation of BH100 in artificial conditions has promoted genetic variability, which resulted in the emergence of sub-strains (Chartone-Souza, 2017).

This single scenario represents a favorable condition to investigate the factors contributing to the genome plasticity of this strain. Although previous studies have suggested the activity of transposons in the plasmids, a large-scale molecular characterization of mobile genetic elements (MGE), involved in the mobilization of drug-resistance genes and virulence factors have not been investigated. Therefore, in this present study, we aimed to assess the full genetic diversity of BH100 sub-strains using WGS to elucidate the factors that might be involved in the mobilization of virulence and drug-resistance genes and their evolutionary relationship with UPEC strains.

## Materials and Methods

### Bacterial Strains

BH100 wild type strain, harboring the pBH100 (conjugative) and pAp (mobilizable) plasmids with antibiotic resistance, was isolated from urine sample of a Brazilian woman from Belo Horizonte in 1974. After isolation, the strain was maintained in tubes containing Lignières medium at room temperature (Parker Hitchens, 1921) and cultures were transferred every 2 years to a new fresh medium. This strain was sequenced in 2014, and referred to in this study as MG2014, and was considered for the investigation of its evolutionary relationship to *E. coli* phylogroups and pathotypes. Two sub-strains were obtained in 1987 by elimination of the plasmids through overnight cultivation at 37°C in nutrient broth containing a subinhibitory concentration of ethidium bromide as previously described (Bouanchaud et al., 1968). The plasmid-cured sub-strains were hereafter referred to as BH100L and BH100N for containing only pBH100 and none of the plasmids, respectively. These sub-strains have been transferred every 6 years to a new fresh medium. All the sub- strains, including BH100 were sequenced lately in 2017 and referred here as MG2017. The sub-strains were selected for investigating the intra-strain activity of mobile elements. All the strains (Supplementary Table 1) were kindly provided by the Microorganism Genetics Laboratory (LGM) of the Federal University of Minas Gerais, Belo Horizonte, Brazil.

### Growth Conditions for Genomic DNA Preparation

The MG2014 strain was grown in 30 mL of BHI medium (HIMEDIA) at 37°C for 18 h and centrifuged at 2,500 g for 15 min. MG2017 sub-strains were grown in 5.0 mL LB medium at 37°C for 18 h. Chloramphenicol (15.0 μg/mL) and 50.0 μg/mL of ampicillin (Sigma) were added in the growth media of the BH100 variants when needed. Genomic DNA was extracted following the protocol of Pacheco et al. (2007) for all bacterial cultures (Pacheco et al., 2007).

### Sequencing of Complete Genomes

BH100 MG2014 was sequenced on the Ion Torrent PGM platform. The construction of a 200 bp fragment library was performed from 1.0 μg DNA using the Ion Xpress™ Plus Fragment Library Kit (Thermo Fisher) as recommended by the manufacturer. The BH100 MG2014 was also sequenced using 3 and 6 kb mate-pair libraries from 10.0 μg of DNA using 5500 SOLiD® Mate-Pair Library Kit, as per Thermo Fisher technical recommendations. All MG2017 sub-strains (BH100, B100L, and BH100N) were sequenced on the Illumina Hi-Seq 2500 platform from 0.1 μg DNA using paired-end libraries (2 × 150 bp) and approximately 500 bp fragments, as recommended by the manufacturer.

### Assembly of Genomes

The quality assessment of the reads was performed using FastQC software. All sub-strains contigs were assembled using Newbler 2.9 and the quality assessment of each assembly was evaluated in QUAST and the best K-mer values were estimated using KmerGenie 1.7023 (Chikhi and Medvedev, 2014). Scaffolding of contigs was performed on CONTIGUATOR software 2.7.3 using the complete genome from *E. coli* 536 (RefSeq: NC_008253.1) as a reference and Gap closure was accomplished via CLC Genome Workbench 7.0 (Galardini et al., 2011). The other bacterial variant reads were submitted to the same pipeline, though using the complete genome *E. coli* BH100 MG2014 as a reference.

### Assembly of Plasmids

For the five plasmids of *E. coli* BH100 sub-strains (NZ_CP024651.1, NZ_CP024652.2, NZ_CP025252.1, NZ_CP025253.1, and CP025139.1) the reads were assembled via PlasmidSpades (Antipov et al., 2016) and the contigs generated were investigated using BLASTn for observation of possible homologous plasmid sequences. Scaffolding of contigs from the pBH100 plasmids was performed in the same manner described above, however using plasmid NR1 (DQ364638.1) as a reference. No gaps were found in pAp contigs.

### Structural and Functional Annotation

The sequences from *E. coli* BH100 sub-strains were annotated using an *in-house* script for annotation transfer from *E. coli* 536. The sequences were also submitted to the RAST automatic annotation tool. Resistance genes were identified using the Comprehensive Antibiotic Resistance Database (CARD) (Alcock et al., 2020). Annotation of insertion sequences (IS) was performed using the ISFinder platform (Siguier et al., 2006). The identification of phage sequences was performed at PHASTER (Arndt et al., 2016). The serotype of BH100 strain was determined via SerotypeFinder 2.0 (Joensen et al., 2015). The Artemis software 16.0 (Carver et al., 2012) was used in the manual curation of annotations based on search results at Uniprot and Pfam databases. Finally, the prediction of the incompatibility groups of each plasmid occurred via PlasmidFinder 1.3 (Carattoli et al., 2014).

### Genomic Synteny Analysis

In order to investigate possible genomic rearrangement and inversion, the synteny between the two plasmids in each strain was investigated through a multiple alignment using Mauve 2.4 (Darling et al., 2004).

### Genomic Islands Prediction

The prediction of genomic islands (GEI) in *E. coli* BH100 variants was performed using the software IslandViewer 4 (Bertelli and Brinkman, 2018) and Genomic Island Prediction Software (GIPSY) (Soares et al., 2016). Virulence factors were predicted through an alignment via BLAST against the virulence factor database (VFDB) (Chen et al., 2005). The *E. coli* K12 MG1655 (RefSeq: NC_000913.3) and *E. coli* 536 (RefSeq: NC_008253.1) strains were used as controls of non-pathogenic and pathogenic strains, respectively. Visualization of GEI was performed in BRIG v0.95 (Alikhan et al., 2011).

### Phylogenetic Analysis

Two approaches were used for phylogenomic analysis, the Phylogenetic Tree Building Service available in the Pathosystems Resource Integration Center (PATRIC) (Wattam et al., 2017) and the PGADB-builder tool (Liu et al., 2016). The complete genome sequence of *E. coli* BH100 MG2014 and other 14 genomes of this species from GenBank representing the phylogroups A, B1, B2, C, D, E, and F were considered in these analysis (Supplementary Table 2). The genome of E. fergusonii ATCC 35469 (NC_011740) was used as an outgroup. For PATRIC's Phylogenetic Tree Building Service, we used the Codon Tree method with 1,000 single-copy gene families. In this pipeline, protein families were identified using PGFams (Davis et al., 2016). The protein sequences are aligned using MUSCLE (Edgar, 2004) and using the codon_align function of BioPython (Cock et al., 2009). The alignments are used as input to estimate the samples phylogeny using the Maximum Likelihood method implemented in RaxML (Stamatakis, 2014), with using 100 rounds of rapid bootstrapping (Stamatakis et al., 2008). Concomitantly, a tree was generated from the whole genome multilocus sequence typing (wgMLST) using the PGADB-builder, which uses a database of alleles from the pan-genome of these strains. Both trees were visualized using iTOL (Letunic and Bork, 2019).

## Results and Discussion

### Sequencing and Assembly

The *E. coli* BH100 sub-strains were sequenced using different Next Generation Sequencing platforms (Ion Torrent and Illumina). A total of 4,269,641 fragment reads, 3,596,096 3 kb-mate-pair reads and 4,518,000 6 kb-mate-pair reads were generated. These genomes have been assembled, annotated, and deposited as complete sequences in GenBank/NCBI (Table 1).

**Table 1:** *E. coli* BH100 variants sequencing and assembly statistics.

**Sub-strain**	**Platform/library**	**Replicon**	**Size**	**Depth**	**Accession number (Genbank)**
BH100 MG2014	Ion torrent/MP and SE	Chromosome	5.072.848	151 ×	NZ_CP024650.2
		Plasmid pBH100-1	107.274	157 ×	NZ_CP024652.2
		Plasmid pAp	14.241	956 ×	NZ_CP024651.1
BH100 MG2017	Illumina Hi-seq/PE	Chromosome	5.131.212	439 ×	NZ_CP025251.1
		Plasmid pBH100-1	105.801	1.464 ×	NZ_CP025253.1
		Plasmid pApR	33.924	1.050 ×	NZ_CP025252.1
BH100L MG2017	Illumina Hi-seq/PE	Chromosome	5.033.217	368 ×	NZ_CP025716.1
		Plasmid pBH100alpha	103.103	1.408 ×	NZ_CP025139.1
BH100N MG2017	Illumina Hi-seq/PE	Chromosome	5.116.036	368 ×	NZ_CP025703.1

*SE, single-end; PE, paired-end; MP, mate-pair*.

### Genomic Synteny of *E. coli* BH100 Sub-strains

**Figure 1:**
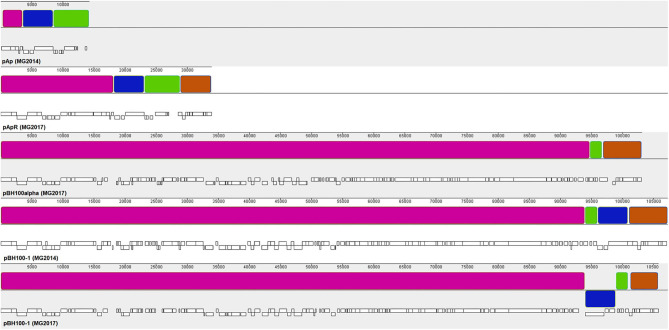
Synteny analysis between the plasmids of *E. coli* BH100 sub-strains. Blocks of the same colors represent homologous nucleotide matches between the plasmids pAp (BH100 MG2014), pApR (BH100 MG2017), pBH100alpha (BH100L MG2017), pBH100 (BH100 MG2017), and pBH100-1 (BH100 MG2014). White bars below each sequence represent the position of CDS. A rearrangement between pApR and pBH100-1 (BH100 MG2017) is indicated by the homologous segments in pink and orange. Other homologous segments in pAp (green and blue blocks) are also found in the plasmids pBH100-1 (BH100 MG2014) and pBH100-1 (BH100 MG2017) suggesting other recombination events. The blue block displayed below the line in pBH100-1 (BH100 MG2017) indicates a genomic inversion at the recombined region. To achieve a better visualization, the original sequences of pAp and pBH100alpha were split in a different genomic position. For the same purpose, the reverse complement sequence of pApR assembly was used and split in a different genomic position.

The synteny analysis of the pBH100 variants performed in Mauve revealed a conserved structure among strains BH100 MG2014, BH100 MG2017, and BH100L MG2017. However, the pApR variant from strain BH100 MG2017, presented a dramatic difference in terms of nucleotide similarity when compared to pAp from BH100 MG2014 (Figure 1). In fact, a portion of ~15 kb from pApR was shown to be identical to a sequence located in pBH100 variants, suggesting a recombination event between the two plasmids in the BH100 MG2017. Other small portions of pAp also seem to be inserted in pBH100 in the MG2014 and BH100 MG2017.

### Differences in Structural and Functional Properties of *E. coli* BH100 Sub-strains

**Figure 2:**
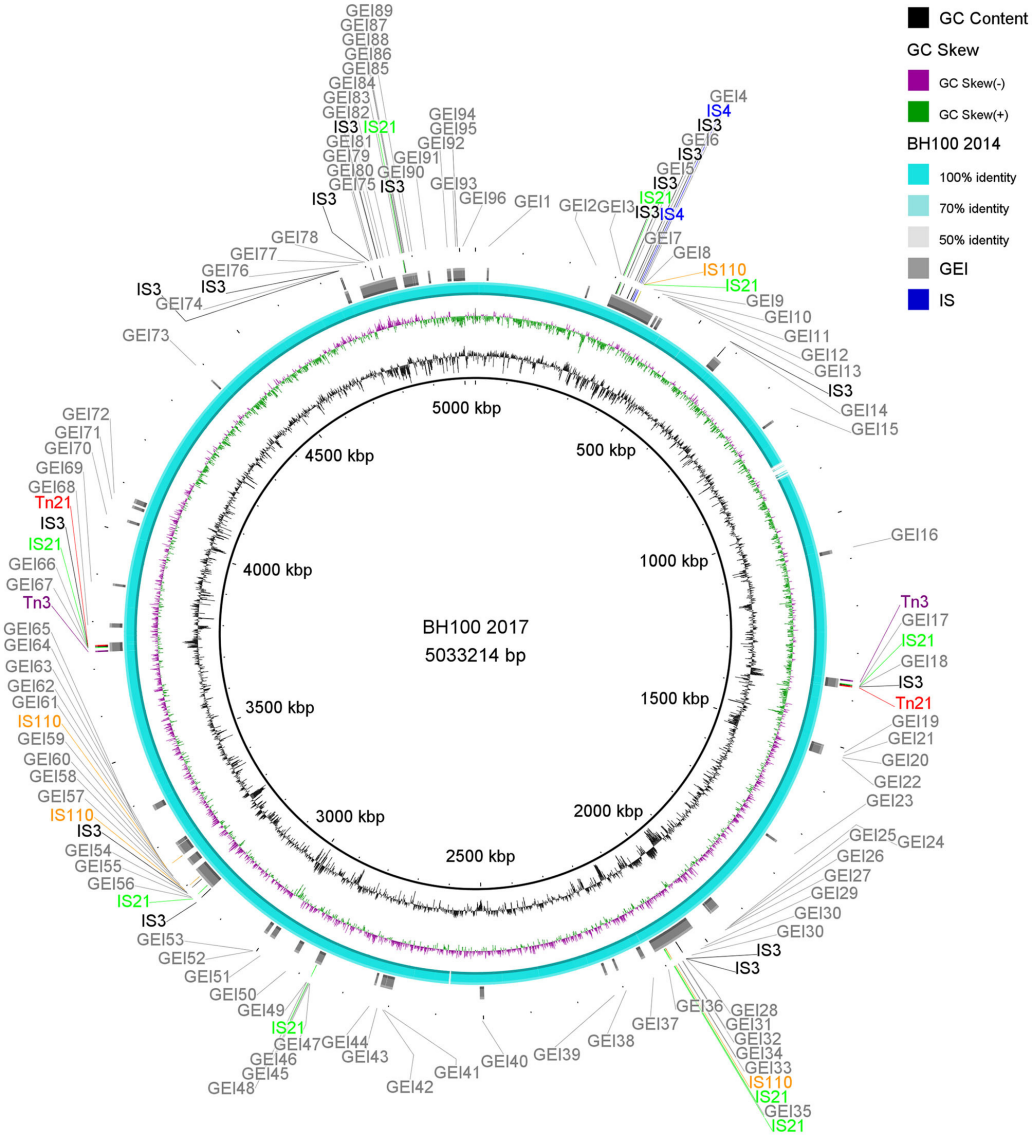
Distribution of IS elements and GEI on *E. coli* BH100L MG2017. An alignment between the genomic sequences of *E. coli* BH100 MG2017 and BH100 MG2014 is shown as indicated by the ring s in light blue (70–100% identity). GEI identified via IslandViewer4 in *E. coli* MG2017 are indicated by the gray bars. The IS elements and other transposases, predicted by the ISfinder tool, are indicated by arrows in green (*IS21*), black (*IS3*), blue (*IS4*), orange (*IS110*), purple (*Tn3*), and red (*Tn21*). GC content is represented by the inner ring in black.

A summary of the structural annotations from *E. coli* BH100 variants chromosomes and plasmids can be seen in Supplementary Tables 3, 4). Distribution of Insertion Sequences (IS) along the chromosomes is illustrated in Figure 2 and Supplementary Figure 1.

In general, our results reveal considerable differences in the number of coding sequences (CDS) and pseudogenes between BH100 variants chromosomes. It has been demonstrated by other studies that IS elements play an essential role in pathogen evolution by providing the sites for genome rearrangements, duplications and deletions (Jackson et al., 2011; Proença et al., 2017). Previous studies suggest *IS3* can function as a mobile promoter in *E. coli* due to the presence of an outward promoter (Charlier et al., 1982). A gene expression approach was not carried out in the present study. In this context, we suggest that a further study comparing the transcriptome profile should be conducted to investigate the promoter activity of *IS3* identified in this work. In summary, these results suggest that the activity of the *IS3* and *IS21* are important factors in the evolution of this strain once they provide genetic variability.

### Drug Resistance Genes and Associated Transposons

**Figure 3:**
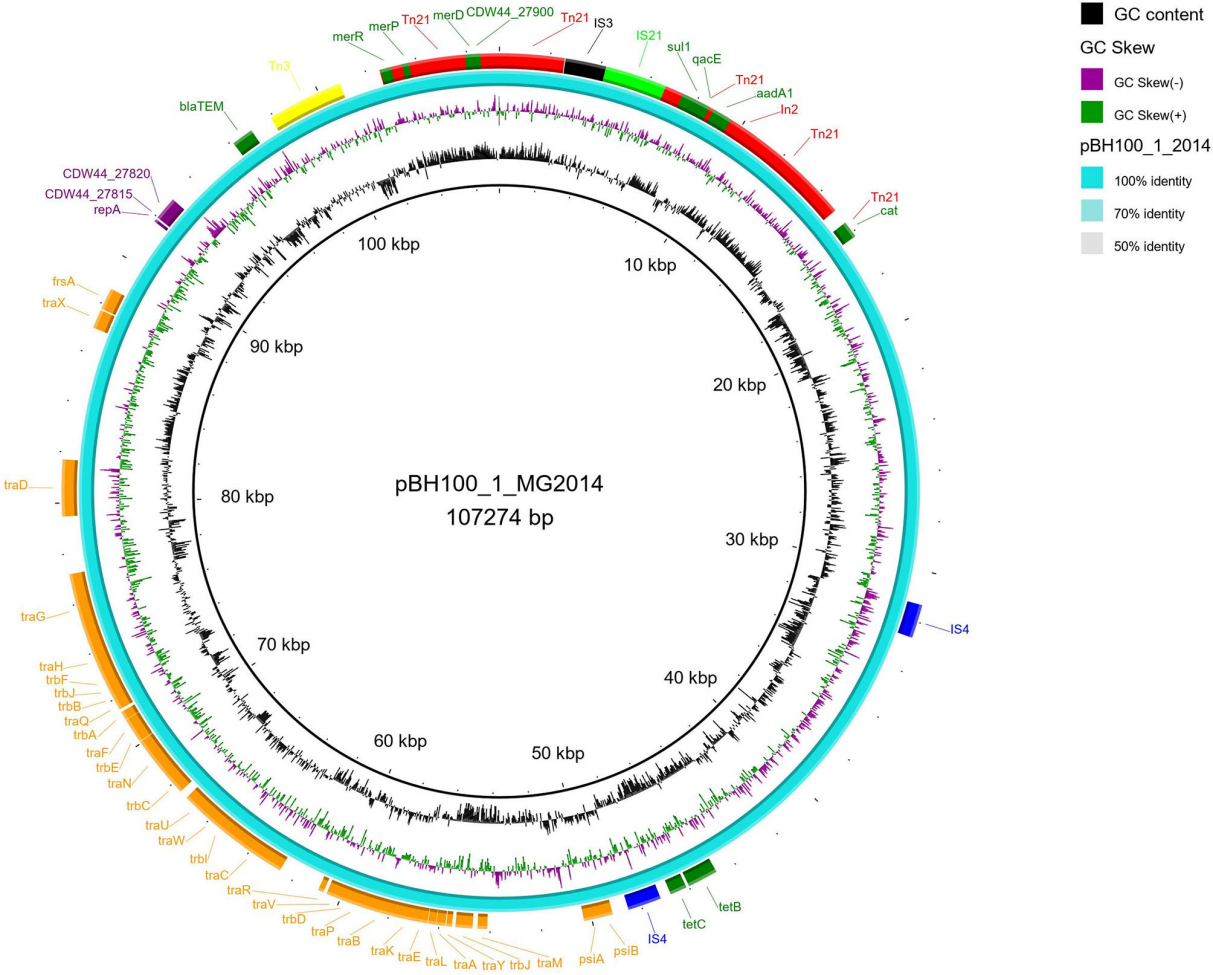
Schematic representation of pBH100-1 from *E. coli* BH100 MG2014. The position of the main genes and features involved in mobilization of MDR in pBH100-1 are represented in the illustration generated in BRIG. The orange and purple bars represent genes involved in the conjugation and replication process, respectively. Antibiotic resistance genes are represented in dark green bars. The IS elements and other transposases, predicted by the ISfinder tool, are indicated by bars in light green (*IS21*), black (*IS3*), blue (*IS4*), yellow (*Tn3*), and red (*Tn21*).

**Figure 4:**
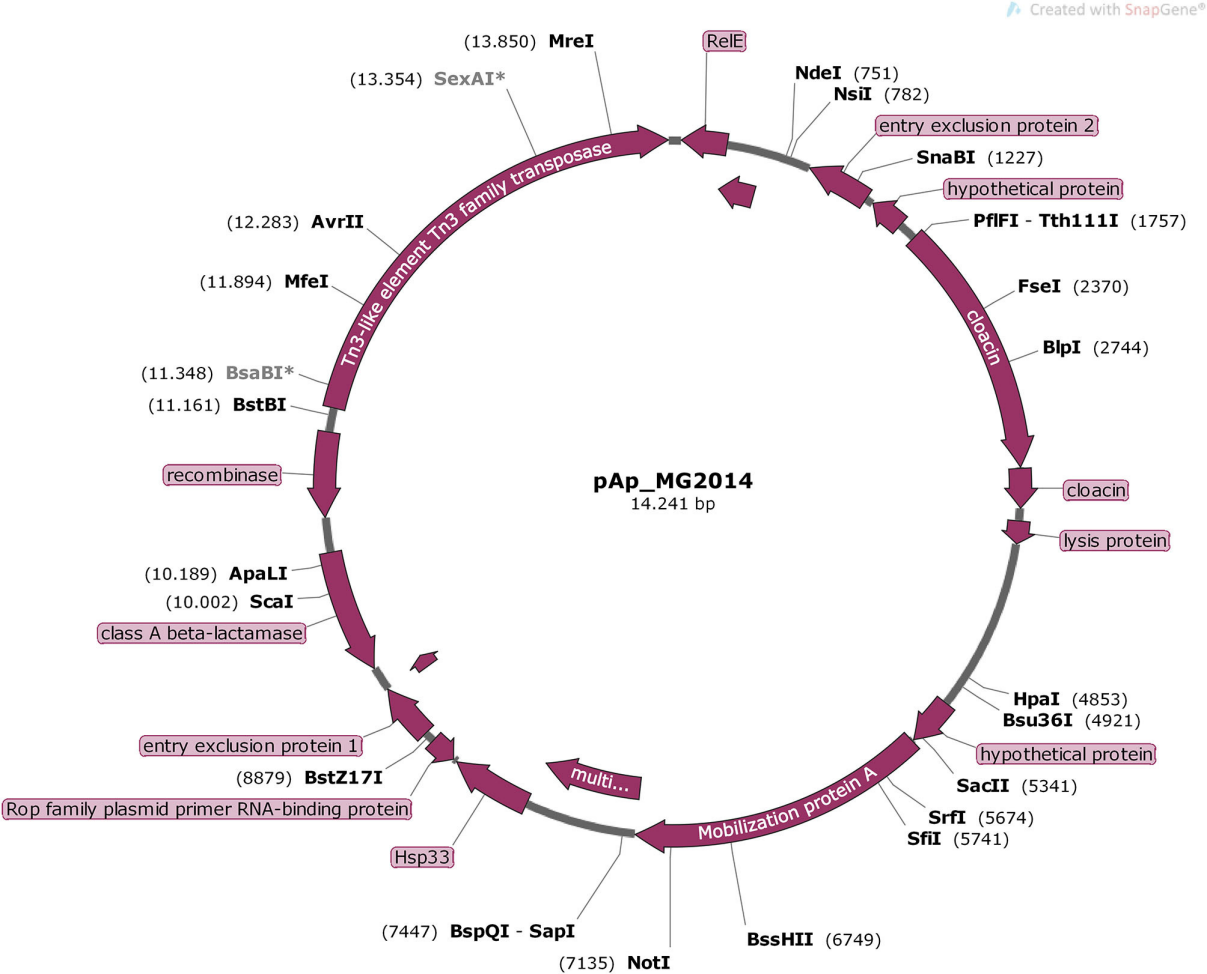
Schematic representation of pAp from *E. coli* BH100 MG2014. The position and orientation of all CDS of pAp annotated in RAST Server are represented in purple arrows.

The genetic map of transposons, and resistance genes, representing the original location and synteny in pBH100 and pAp from BH100 MG2014 are illustrated in Figures 3, 4. Additional information for pBH100 is available in the Supplementary Table 5.

**Figure 5:**
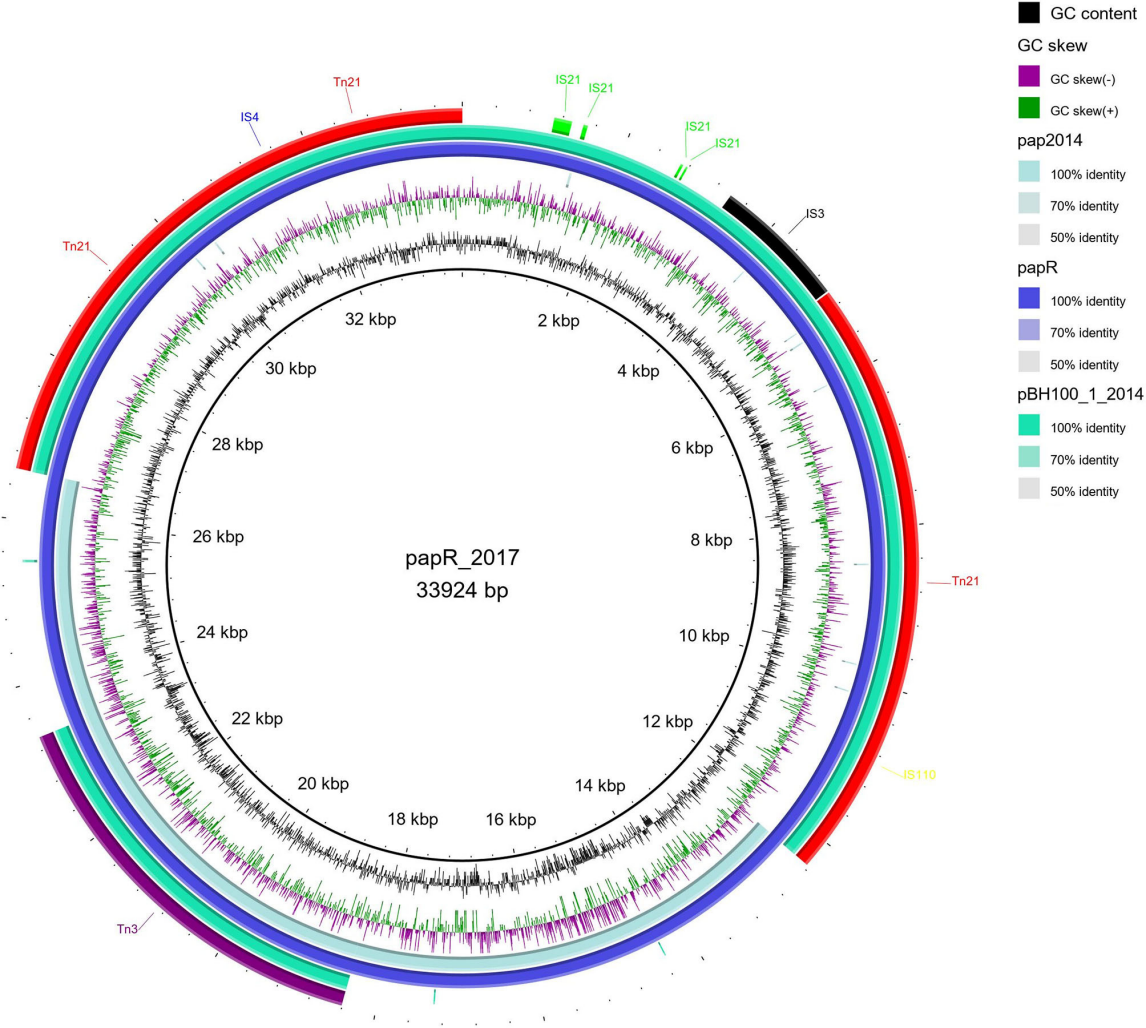
Distribution of IS elements in pApR from *E. coli* BH100 MG2017. An alignment (70–100% identity) between pApR sequence (BH100 MG2017) and the plasmids pAp (BH100 MG2014) and pBH100-1 (BH100 MG2014), performed in BRIG. is represented by the rings in emerald green and blue colors. The IS elements and other transposases, predicted by the ISfinder tool, are indicated by arrows in green (*IS21*), black (*IS3*), blue (*IS4*), yellow (*IS110*), purple (*Tn3*), and red (*Tn21*). GC content is represented by the inner ring in black.

Among the MGE observed, we highlight *Tn21*, which was detected in all variants of pBH100. This transposon contains the class 1 integron, *In2*, and it is possibly responsible for the mobilization of the *mer* operon and the streptomycin resistance gene, *aadA1*. *Tn21* was first described in the R100 plasmid, also known as NR1 from *Shigella flexneri*, which also determines resistance to inorganic mercury. Other studies have reported a conserved structure of this transposon in many other R plasmids from different Gram-negative bacteria, suggesting it is widely distributed (Clennel et al., 1995; Kiyono et al., 2009). The presence of *In2* has been considered one of the most important factors contributing to MDR wide-spread in Gram-negative pathogens as they have the ability to capture and eventually accumulate gene cassettes that confer adaptive advantages (Chang et al., 2009; Ahangarzadeh Rezaee et al., 2012; Firoozeh et al., 2019). In this study, we show that a Tn21 was detected in the recombined region (locus tag BH100Bp05113) of pApR (NZ_CP025252.1) from BH100 MG2017 (Figure 5 and Supplementary Figure 2). Furthermore, IS21 and IS3 transposases were found located upstream of this transposon, suggesting they could play a role in the mobilization of MDR genes in *E. coli* BH100 strain (Figure 5). Intriguingly, the presence of *Tn21* has also been detected in the *E. coli* BH100L MG2017 chromosome but not in the remaining replicons, indicating that it was probably transferred from pBH100 (Figure 2).

All pAp variants presented *Tn3*, which carries the gene *bla*, encoding the β-lactamase TEM-1 type (Gerlach and Wiedemann, 1985). Surprisingly, *Tn3* was also found in pBH100-1 from both BH100 MG2014 and BH100 MG2017 but not in the pBH100alpha from the pAp-cured strain BH100L MG2017. Thus, this finding suggests *Tn3* was disseminated from pAp to other replicons.

The description of other transposons associated with MDR genes which were not present in rearrangements can be found in the Supplementary Material. In addition to the resistance genes located in the plasmids, analysis performed by CARD and UniProtKB/Swiss-Prot databases in this study predicted up to 18 drug transmembrane transport genes and 29 transcriptional regulators associated with multidrug resistance, including fluoroquinolone class in the chromosome of all BH100 sub-strains (80–100% identity) (Supplementary Table 6).

Therefore, our findings show that several resistance genes from *E. coli* BH100 are contained in functional transposable elements as we confirm their activity as being involved in genomic rearrangements, thus representing the potential ability of this strain to mobilize antibiotic resistance genes.

### Genomic Islands Prediction

The genomes from *E. coli* BH100 sub-strains presenting rearrangements were analyzed for the prediction of GEI (Figure 2 and Supplementary Figure 1). In total, around 80 islands were predicted in BH100 variants. Moreover, we predicted 72 transposase genes in BH100 MG2014 GEI, in which the majority, represented by 16 and 12 genes, were classified into *IS3* and *IS21* families, respectively. The GEI distribution was shown to be conserved among the strains, though BH100L MG2017 presented two integrated sequences of *Tn21*, associated with Mer *operon* and streptomycin resistance genes, in 2 different islands. This result suggests that the genetic variability promoted by IS21 and IS3 could have an important role in the formation of resistance islands. Interestingly, a recent study has reported UPEC strains in Mexico containing Tn21 with similar MDR cassette, but integrated in resistance islands in the chromosome (Paniagua-Contreras et al., 2019). In this context, our work supports the hypothesis that pBH100 and other related R100 plasmids might be involved for MDR spreading through the development of Resistance Islands alternatively to conjugation mechanisms.

**Figure 6:**
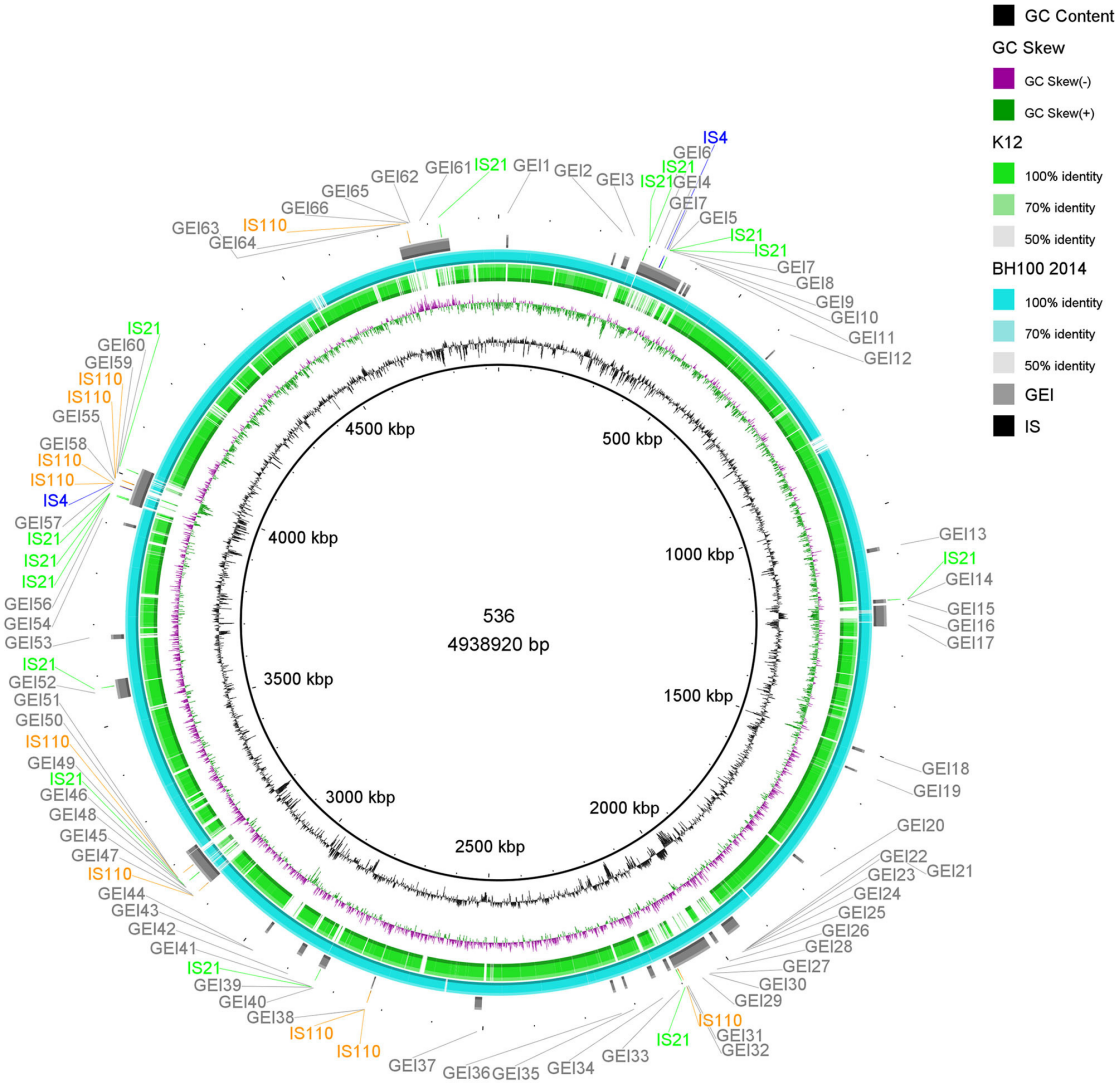
Distribution of IS elements and GEI on *E. coli* 536. An alignment (70–100% identity) between the genomic sequences of *E. coli* 536 and the strains BH100 MG2014 and K12, performed in BRIG, is represented by the rings in light blue and green colors. GEI identified via IslandViewer4 in *E. coli* 536 are indicated by the gray bars. The IS elements, predicted by the ISfinder tool, are indicated by arrows in green (*IS21*), blue (*IS4*), and orange (*IS110*). GC content is represented by the inner ring in black.

In order to identify genes coding for uropathogenic virulence factors, the results of BH100 MG2014 were compared to the non-pathogenic *E. coli* K-12 MG1655 and the UPEC 536, as shown in Figure 6 and Supplementary Figures 3, 4). This analysis shows the majority of GEI are shared between *E. coli* BH100 and 536 but are not present in the non-pathogenic K12 strain, suggesting these GEI are probably pathogenicity islands (PAI). The gene content related to virulence of those possible PAI are discussed below.

An important uropathogenic virulence factor is the hemolysin toxin, which causes epithelial cell exfoliation and promotes the dissemination of the pathogen (Justice and Hunstad, 2012). Our data reveal the presence of eight genes (two *hlyABCD* operons) involved in transport, activation, and secretion of hemolysin both in the BH100 strain and in the *E coli* 536 while, as expected, no hemolysin related gene was found in K-12. Recent studies have reported iron uptake genes as being vital in the pathogenesis of UPEC strains to overcome the scarcity of iron (Robinson et al., 2018; Bauckman et al., 2019). Interestingly, three siderophore genes were found in the *E. coli* strains 536 and BH100 MG2014 but none in the K-12.

In summary, the presence of IS3 and IS21 in the predicted genomic islands containing genes associated with virulence in *E. coli* BH100 suggest their potential role in providing mobility of virulence factors.

### Evolutionary Relationships of *E. coli* BH100 Replicons With UPEC and Other MDR Bacteria

**Figure 7:**
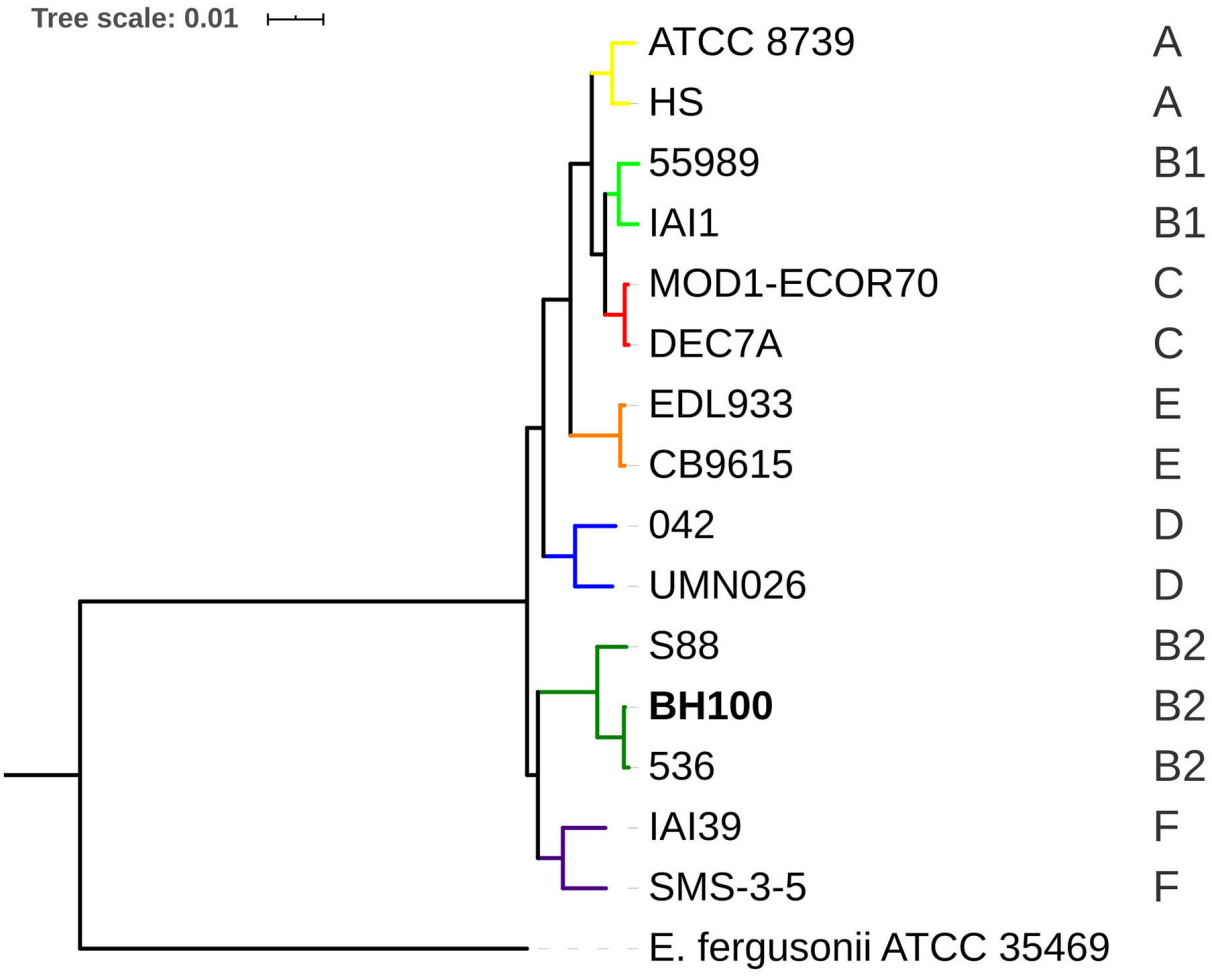
Phylogenomic tree of *E. coli* BH100 and GenBank sequences inferred by PATRIC. The uppercase letters (A, B1, B2, C, D, E, and F) in the right corner indicate *E. coli* phylogroups. The bootstrap values ranged from 92 to 100%.

Both phylogenomic approaches revealed *E. coli* BH100 is clustered together with the 536 and S88 strains representing the B2 phylogroup thus showing the robustness of the methods used in this study (Figure 7 and Supplementary Figure 5). Furthermore, this result supports that BH100 might be considered as UPEC pathotype once the *E. coli* 536 genome is a model for the genetic basis of uropathogenic virulence (Dobrindt et al., 2001). Though some differences between the two strains can be highlighted as indicated by the serotype analysis revealing that BH100 (O6: H31) does not contain the capsular antigens, which are present in the 536 strain (O6: K15: H31) (Wiles et al., 2008). Moreover, when contemplating the *IS3* family, it has been rarely reported in *E. coli* 536 (Terlizzi et al., 2017). Therefore, these differences might suggest the acquisition of genetic features, including MGE from different sources.

To determine the incompatibility groups of *E. coli* BH100 plasmids, the nucleotide sequence was analyzed on PlasmidFinder. These results show that pBH100 and pAp belong to IncFII and ColRNAI groups (100% identity), respectively. Interestingly, plasmids from these incompatibility groups have not been reported in 536 strain, suggesting BH100 may have acquired them from another strain. The alignment via BLASTn against Non-redundant sequences of the NCBI database indicates that the NR1 sequence (GenBank: DQ364638.1), a representative plasmid of IncFII, is very similar to pBH100alpha from *E. coli* BH100 MG2014 (99.74% identity, 92% coverage, E value: 0.0). The nucleotide sequence of *Tn21* present in pBH100 proved to be identical to that described in plasmid NR1, which carries the *In2* associated with the *mer* operon. Previous studies have reported the wide distribution of *Tn21*/*In2* in enterobacteria, might be explained by the exposure of toxic metals in agricultural and industrial practice at the same time when the clinical use of antibiotic drugs was rapidly increasing (Trieu-Cuot et al., 1993; Nascimento and Chartone-Souza, 2003).

Regarding pAp from *E. coli* BH100 MG2014, it was possible to observe a high similarity to many *Klebsiella sp*. plasmids through the alignment via BLASTn, especially pNJST258N4 from *Klebsiella pneumoniae* 30660/NJST258_1 (GenBank DQ298019.1) (99.93% identity, 92% coverage, E value: 0.0). The closest *E. coli* plasmid sequence revealed to be pECAZ161_KPC (GenBank: CP019010.1). However, the significant difference is the presence of the *Klebsiella pneumoniae* carbapenemase enzyme gene located in pECAZ161_KPC. This result is very interesting, as carbapenemases have appeared over the last 18 years as the most clinically relevant antimicrobial resistance in *Enterobacteriaceae* (Stoesser et al., 2017). Although the present study does not support the direction of genetic flow between these strains, we suppose that BH100 might have either the ability to acquire this plasmid from *Klebsiella* sp. or vice versa.

## Conclusion

This study has described *E. coli* BH100 genetic diversity, especially concerning functional mobile elements associated with MDR and virulence genes potentially related to uropathogenesis. To our knowledge, it is the first time that the *IS3* family has been associated with UPEC pathotype. Furthermore, our results have shown that conserved regions of *E. coli* BH100 are shared not only with UPEC strains but also with other MDR *Enterobacteriaceae*. In this context, we highlight the importance of future studies further to investigate the evolutionary role of *IS3* in UPEC strains and other enterobacteria.

## Data Availability Statement

The datasets presented in this study can be found in online repositories. The names of the repository/repositories and accession number(s) can be found in the article/Supplementary Material.

## Author Contributions

RC performed structural and functional bioinformatic analysis of the E. coli strains, interpreted the data and was the major contributor in writing the manuscript. FA was responsible for bioinformatic analysis, interpretation of data. MC performed the phylogenomic analysis and contributed to manuscript writing. EC-S and AN was responsible for ceding the E. coli BH100 strains and contributed to data interpretation. LJ performed DNA extraction. AZ contributed to manuscript writing and data interpretation. BB was responsible for samples sequencing and manuscript writing. DB, PG, and AG-N contributed to data interpretation and writing the manuscript. HF performed sample sequencing and manuscript writing. SS was responsible for genomic island prediction and explanation of the analysis. RR and AP contributed to the analysis and interpretation of genomic data. VA provided interpretation of genomic data and was a major contributor to the revision of the manuscript.

## Conflict of Interest

The authors declare that the research was conducted in the absence of any commercial or financial relationships that could be construed as a potential conflict of interest.
