# Supplementary material for: Genomic Characterization of Multidrug-Resistant Escherichia coli BH100 Sub-strains
Source: Front Microbiol. 2021 Jan 8;11:549254. doi: 10.3389/fmicb.2020.549254 (PMC7874104; doi:10.3389/fmicb.2020.549254)
Supplement: Supplementary file 1 [file Table_1.DOC]

**Table S1. Bacterial strains and plasmids used in this work**

| **Substrain** | **Plasmids** | **Antibiotic Resistance** |
| --- | --- | --- |
| *E. coli* BH100 MG2014 | pBH100-1, pAp | HgKnCmTcSmAp |
| *E. coli* BH100 MG2017 | pBH100-1, pApR | HgKnCmTcSmAp |
| *E. coli* BH100L subtr. MG2017 | pBH100alpha | HgKnCmTcSm |
| *E. coli* BH100N subtr. MG2017 | - | - |
