# Supplementary material for: Genomic Characterization of Multidrug-Resistant Escherichia coli BH100 Sub-strains
Source: Front Microbiol. 2021 Jan 8;11:549254. doi: 10.3389/fmicb.2020.549254 (PMC7874104; doi:10.3389/fmicb.2020.549254)
Supplement: Supplementary file 2 [file Table_2.DOC]

**Table S2. Genomes from GenBank representing the *E. coli* phylogroups A, B1, B2, C, D, E and F.**

| **Strain** | **RefSeq Accessions** | **Phylogenetic Group** | **PATRIC ID** |
| --- | --- | --- | --- |
| *E. coli* EDL933 | NC_002655,NC_007414 | E | 155864,8 |
| *E. coli* 042 | NC_017626.1,NC_017627.1 | D | 216592,3 |
| *E. coli* HS | NC_009800 | A | 331112,6 |
| *E. coli* 536 | NC_008253 | B2 | 362663,9 |
| *E. coli* SMS-3-5 | NC_010498,NC_010486,NC_010487,NC_010488,NC_010485 | F | 439855,1 |
| *E. coli* ATCC 8739 | NC_010468 | A | 481805,6 |
| *E. coli* MOD1-ECOR70 | GCF_003333475.1 | C | 562,33307 |
| *E. coli* IAI1 | NC_011741 | B1 | 585034,5 |
| *E. coli* S88 | NC_011742,NC_011747 | B2 | 585035,6 |
| *E. coli* 55989 | NC_011748 | B1 | 585055,8 |
| *E. coli* UMN026 | NC_011751,NC_011749,NC_011739 | D | 585056,7 |
| *E. coli* IAI39 | NC_011750 | F | 585057,6 |
| *E. coli* CB9615 | NC_013941,NC_013942 | E | 701177,3 |
| *E. coli* DEC7A | GCF_000249695.1 | C | 868165,3 |
| *E. fergusonii*  ATCC 35469 | NC_011740,NC_011743 | outgroup | 585054,5 |
