# Supplementary material for: Genomic Characterization of Multidrug-Resistant Escherichia coli BH100 Sub-strains
Source: Front Microbiol. 2021 Jan 8;11:549254. doi: 10.3389/fmicb.2020.549254 (PMC7874104; doi:10.3389/fmicb.2020.549254)
Supplement: Supplementary file 3 [file Table_3.DOCX]

**Table S3. Annotation statistics of chromosomes of *E. coli* BH100 substrains.**

| **Chromosomes** | **CDS** | **Pseudogenes** | **tRNA** | **rRNA** | **%GC** |
| --- | --- | --- | --- | --- | --- |
| **BH100 MG2014** | 4.971 | 68 | 84 | 22 | 50.38 |
| **BH100 MG2017** | 5.080 | 27 | 82 | 22 | 50.71 |
| **BH100L MG2017** | 4.827 | 85 | 81 | 22 | 50.53 |
| **BH100N MG2017** | 4.981 | 79 | 93 | 22 | 50.44 |
