# Supplementary material for: Genomic Characterization of Multidrug-Resistant Escherichia coli BH100 Sub-strains
Source: Front Microbiol. 2021 Jan 8;11:549254. doi: 10.3389/fmicb.2020.549254 (PMC7874104; doi:10.3389/fmicb.2020.549254)
Supplement: Supplementary file 4 [file Table_4.DOCX]

**Table S4. Annotation statistics of plasmids of *E. coli* BH100 substrains.**

| **Plasmids** | **CDS** | **Pseudogenes** | **%GC** | **Size (bp)** |
| --- | --- | --- | --- | --- |
| **pAp (MG2014)** | 17 | 0 | 53,33 | 14,241 |
| **pApR (MG2017)** | 36 | 0 | 57,3 | 33,924 |
| **pBH100alpha (MG2017)** | 136 | 0 | 52,02 | 103,103 |
| **pBH100-1 (MG2014)** | 133 | 0 | 51,91 | 107,274 |
| **pBH100-1 (MG2017)** | 140 | 0 | 52,02 | 105,801 |
