# Supplementary material for: Genomic Characterization of Multidrug-Resistant Escherichia coli BH100 Sub-strains
Source: Front Microbiol. 2021 Jan 8;11:549254. doi: 10.3389/fmicb.2020.549254 (PMC7874104; doi:10.3389/fmicb.2020.549254)
Supplement: Supplementary file 7 [file Data_Sheet_1.DOCX]

Two other possible resistance genes were observed between the *mer* operon and the *aadA1* gene. The potential resistance to sulfonamides is encoded by the *sul1* gene that synthesizes the enzyme dihydropteroate synthase (Razavi et al., 2017). The other gene, *qacEΔ1*, is located immediately downstream and has been described as encoding the Quaternary ammonium compound-resistance protein. It is known that compounds containing four ammonium groups in their composition are associated with several bactericides and disinfectants found on the market (Jiang et al., 2017).Our data suggest *Tn21* is inserted in another transposon, Tn9, which carries the *cat* gene encoding chloramphenicol acetyltransferase (Trieu-Cuot et al., 1993). *Tn10* was exclusively found in pBH100 variants, containing the sequence of the *operon* that confers resistance to tetracycline (Chopra and Roberts, 2001). Within this transposon sequence, it was also verified a feature annotated as *Tn5*-like, carrying the kanamycin resistance gene *aphA1,* flanked by *IS5* (Li et al., 2015). Interestingly, insertion sequences from *IS5* family were also found on the BH100 chromosome MG2014, suggesting the possible transference of *aphA1* to the chromosome.
