# Supplementary material for: Genomic Characterization of Multidrug-Resistant Escherichia coli BH100 Sub-strains
Source: Front Microbiol. 2021 Jan 8;11:549254. doi: 10.3389/fmicb.2020.549254 (PMC7874104; doi:10.3389/fmicb.2020.549254)
Supplement: Supplementary file 8 [file Data_Sheet_2.DOC]

**Supplementary material has been published at the public repository 10.6084/m9.figshare.12084795**

**Supplementary Figures and Tables legends**

**Table S1. Bacterial strains and plasmids used in this work.**

**Table S2. Genomes from GenBank representing the *E. coli* phylogroups A, B1, B2, C, D, E and F.**

**Table S3. Annotation statistics of chromosomes of *E. coli* BH100 sub-strains.**

**Table S4. Annotation statistics of plasmids of *E. coli* BH100 sub-strains.**

**Table S5. pBH100-1 annotated CDS and features.**

**Table S6. Prediction of resistance genes in *E. coli* BH100 MG2014.**

**Figure S1. Distribution of IS elements and GEI on *E. coli* BH100L MG2014.**

**Figure S2. Tn21 recombination in pApR.**

**Figure S3. Distribution of IS elements and GEI on *E. coli* K12.**

**Figure S4. Heatmap of predicted virulence genes in the six *E. coli* strains genomes.**

**Figure S5. Phylogenetic tree of *E. coli* BH100 and GenBank sequences inferred by MLST.**
