# Supplementary figures and images for: Genomic Characterization of Multidrug-Resistant Escherichia coli BH100 Sub-strains
Source: Front Microbiol. 2021 Jan 8;11:549254. doi: 10.3389/fmicb.2020.549254 (PMC7874104; doi:10.3389/fmicb.2020.549254)

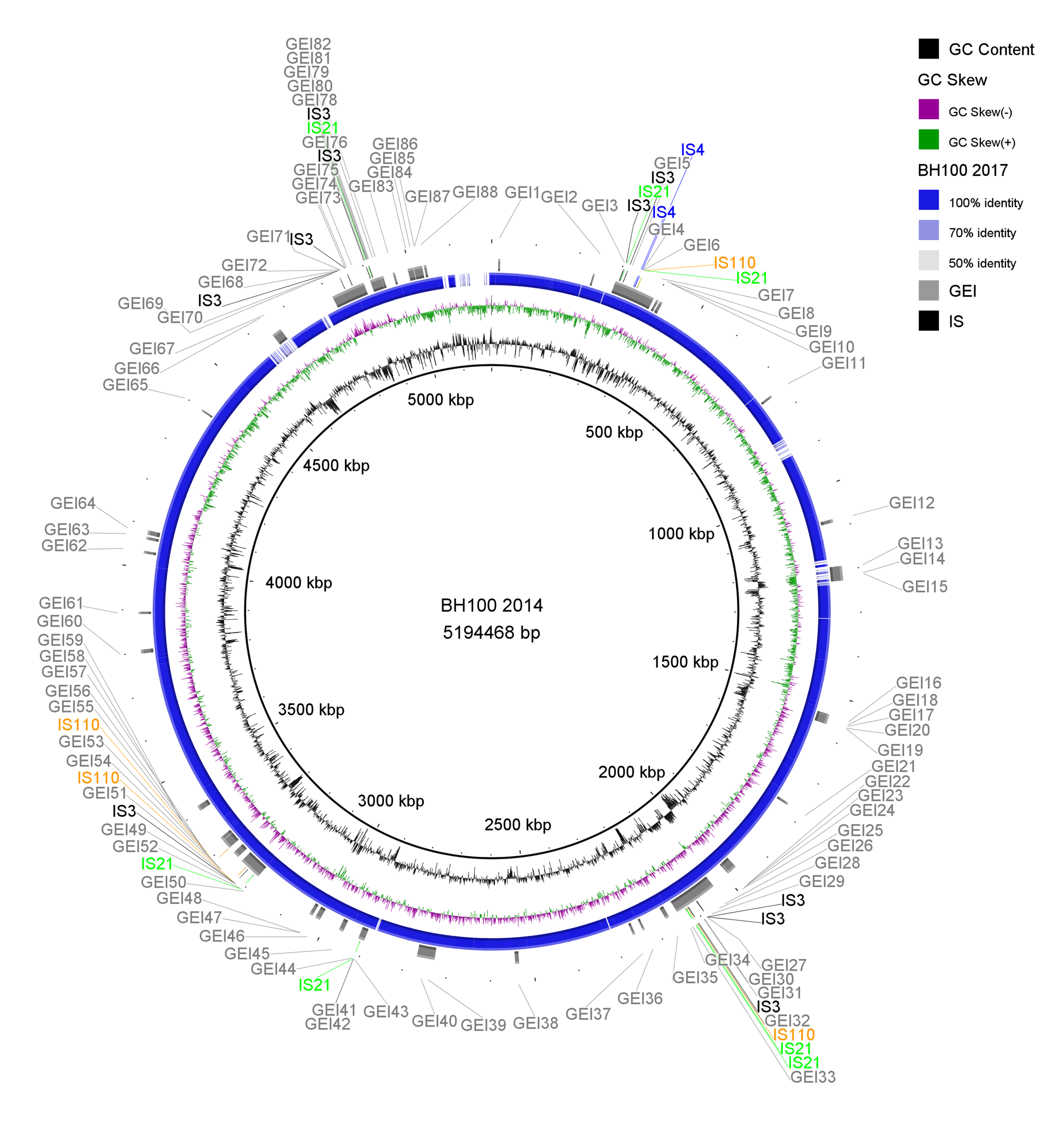

Supplement: Supplementary file 9 [file Image_1.TIF]

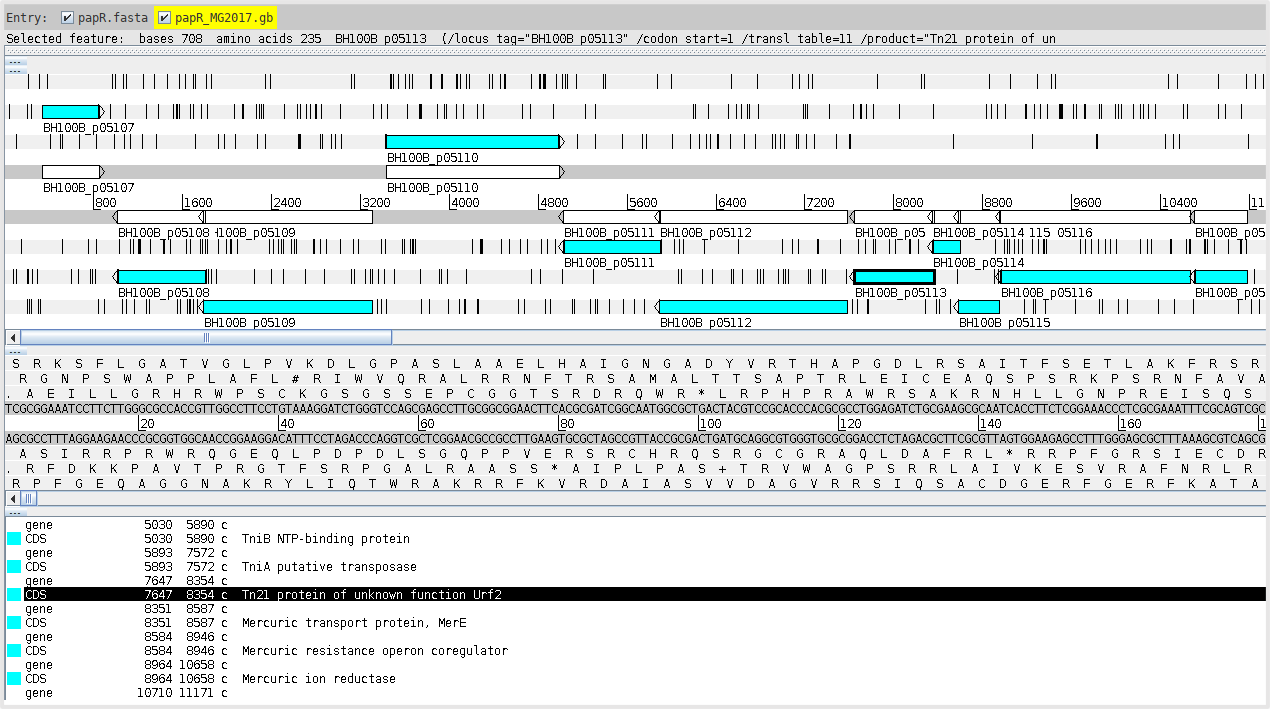

Supplement: Supplementary file 10 [file Image_2.TIF]

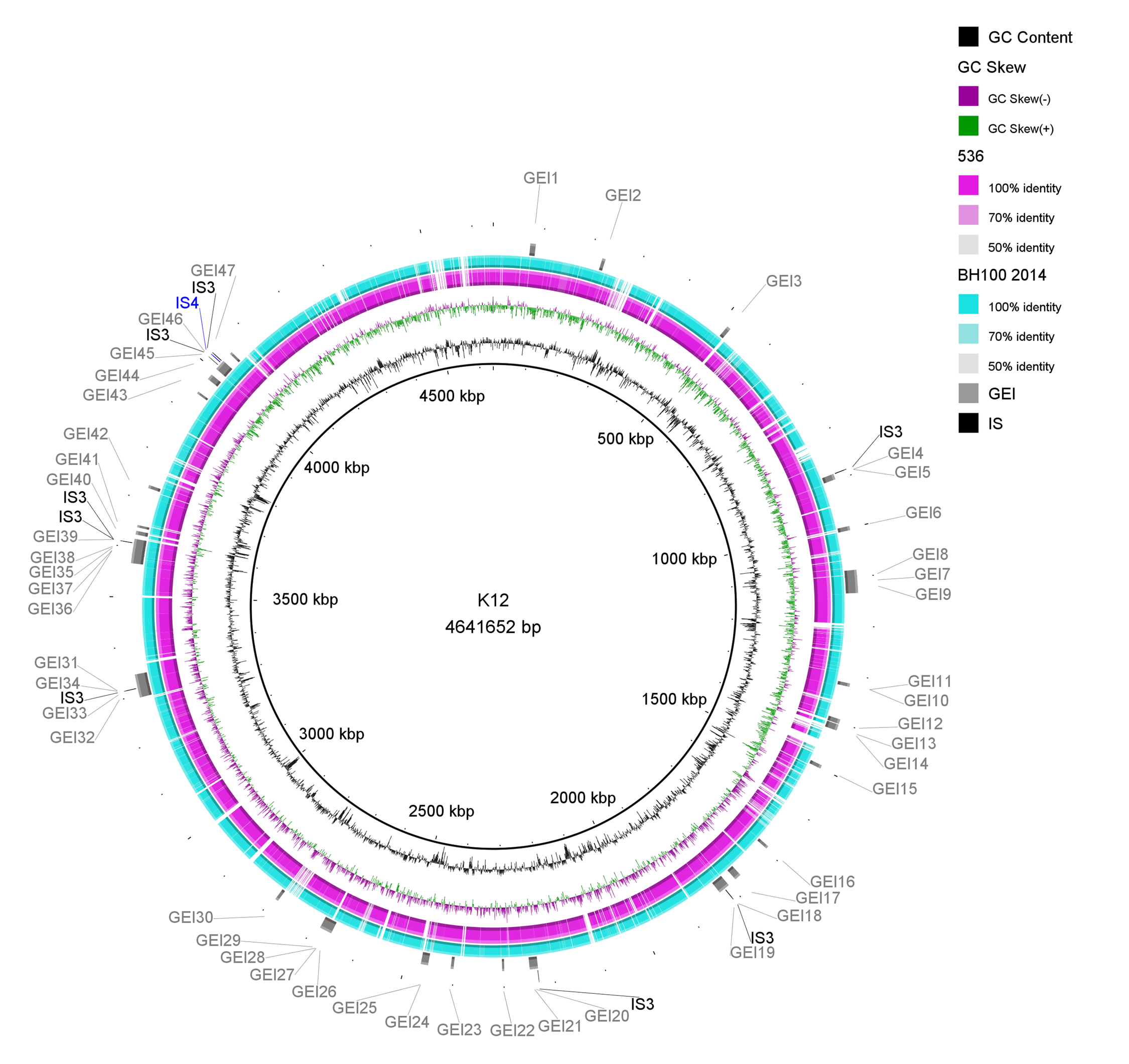

Supplement: Supplementary file 11 [file Image_3.TIF]

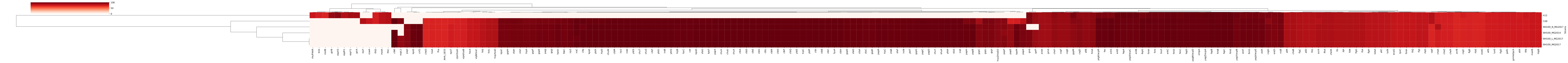

Supplement: Supplementary file 12 [file Image_4.JPEG]

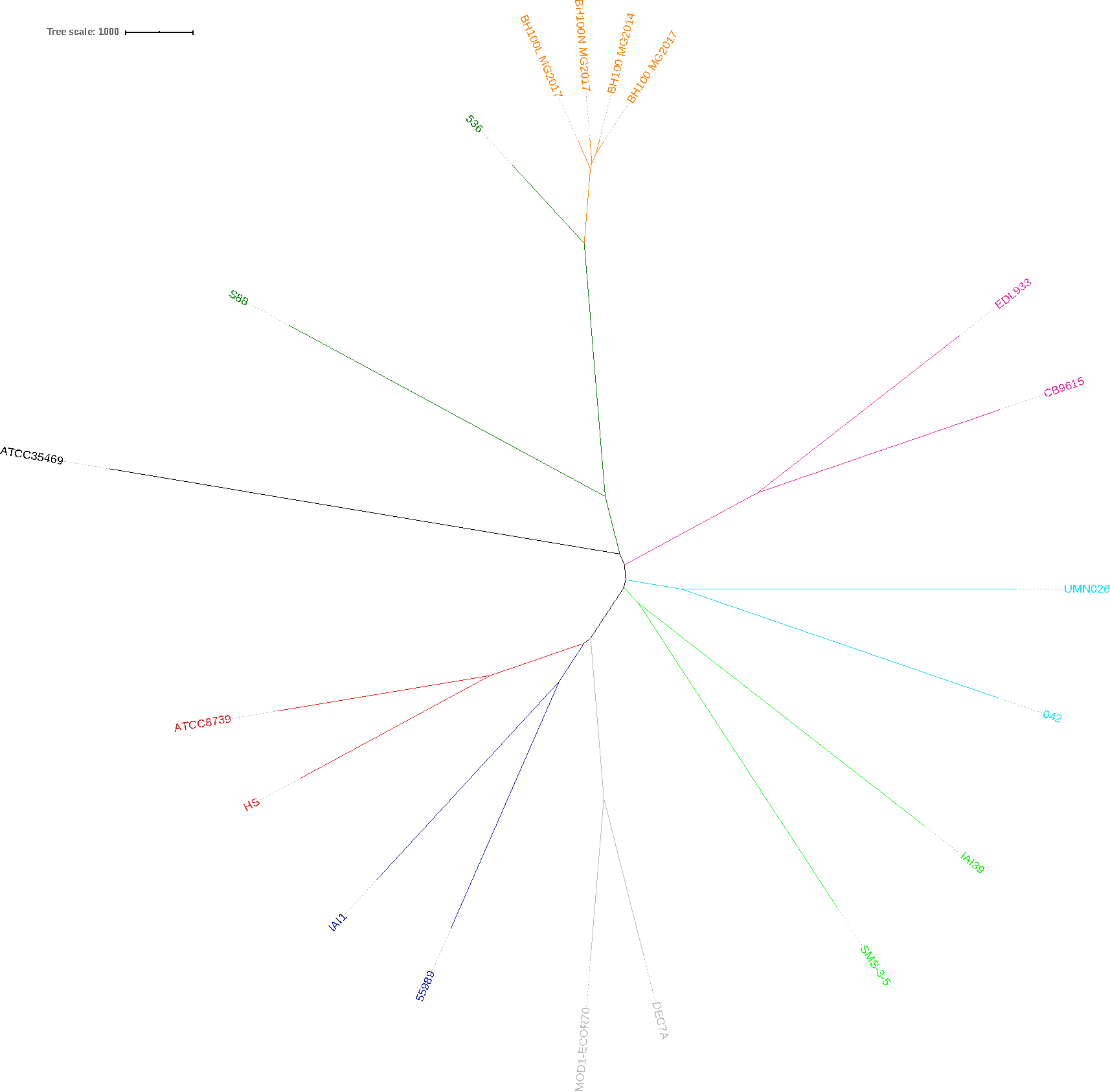

Supplement: Supplementary file 13 [file Image_5.JPEG]
